# Interphase cytogenetics of multicentric renal cell tumours confirm associations of specific aberrations with defined cytomorphologies

**DOI:** 10.1054/bjoc.1999.1126

**Published:** 2000-03-21

**Authors:** B K Amo-Takyi, C Mittermayer, K Günther, S Handt

**Affiliations:** Institute of Pathology, Medical Faculty, Aachen University of Technology, Pauwelsstrasse 30, Aachen, 52057, Germany

**Keywords:** chromosomal in situ hybridization, multicentric renal cell tumours, chromosomal aberrations, tumour size, stage and grade

## Abstract

To demonstrate associations of certain chromosomal aberrations with defined renal cell tumour (RCT) subtypes, we analysed 239 tumour nephrectomy cases for specimens with multicentric tumours. Chromosomal in situ hybridization was then performed on 15 cases with 34 foci (16 conventional renal cell carcinomas (RCCs), and 18 papillary RCTs (11 carcinomas and seven adenomas) for specific chromosomal aberrations, using α-satellite probes for chromosomes 3, 7 or 17. Particular preference was given to cases which had separate foci with different cytomorphologies. Furthermore, we compared aberrations in relation to tumour size, stage, grade and between different foci in a specimen. Thirty-four cases had multiple tumours. Forty-seven per cent of the multicentric tumours were conventional RCCs and 53% papillary RCTs (against 83% solitary conventional RCCs and 5% solitary papillary RCTs). Three conventional RCCs sized 8 mm (G3), 13 cm (pT2, G2) and 15 cm (pT3b, G3), respectively, revealed monosomy 3, and 13 were disomic. Seventeen papillary RCTs (11 carcinomas and six adenomas) displayed trisomy 17, irrespective of size or grade. Four papillary carcinomas and six papillary adenomas had trisomy 7, and the rest (seven papillary carcinomas and one papillary adenoma) revealed disomy 7. In conclusion, papillary RCTs were tendentially multicentric. Although specific for conventional RCCs heedless of size, monosomy 3 was only observed in high-grade and/or advanced tumours. Trisomy 17 was only detectable in papillary RCTs irrespective of tumour state, showing increased copies with tumour growth. Papillary RCTs also appeared to lose some copies of chromosome 7 with tumour progress, possibly reflecting malignancy. © 2000 Cancer Research Campaign


*British Journal of Cancer* (2000) **82**(8), 1407–1414
© 2000 Cancer Research Campaign
DOI: 10.1054/ bjoc.1999.1126, available online at http://www.idealibrary.com on IDEAL®

### Interphase cytogenetics of multicentric renal cell tumours confirm associations of specific aberrations with defined cytomorphologies

**BK Amo-Takyi, C Mittermayer, K Günther and S Handt**

Institute of Pathology, Medical Faculty, Aachen University of Technology, Pauwelsstrasse 30, 52057 Aachen, Germany

**Summary** To demonstrate associations of certain chromosomal aberrations with defined renal cell tumour (RCT) subtypes, we analysed 239 tumour nephrectomy cases for specimens with multicentric tumours. Chromosomal in situ hybridization was then performed on 15 cases with 34 foci (16 conventional renal cell carcinomas (RCCs), and 18 papillary RCTs (11 carcinomas and seven adenomas) for specific chromosomal aberrations, using $\alpha$-satellite probes for chromosomes 3, 7 or 17. Particular preference was given to cases which had separate foci with different cytomorphologies. Furthermore, we compared aberrations in relation to tumour size, stage, grade and between different foci in a specimen. Thirty-four cases had multiple tumours. Forty-seven per cent of the multicentric tumours were conventional RCCs and 53% papillary RCTs (against 83% solitary conventional RCCs and 5% solitary papillary RCTs). Three conventional RCCs sized 8 mm (G3), 13 cm (pT2, G2) and 15 cm (pT3b, G3), respectively, revealed monosomy 3, and 13 were disomic. Seventeen papillary RCTs (11 carcinomas and six adenomas) displayed trisomy 17, irrespective of size or grade. Four papillary carcinomas and six papillary adenomas had trisomy 7, and the rest (seven papillary carcinomas and one papillary adenoma) revealed disomy 7. In conclusion, papillary RCTs were tendentially multicentric. Although specific for conventional RCCs heedless of size, monosomy 3 was only observed in high-grade and/or advanced tumours. Trisomy 17 was only detectable in papillary RCTs irrespective of tumour state, showing increased copies with tumour growth. Papillary RCTs also appeared to lose some copies of chromosome 7 with tumour progress, possibly reflecting malignancy. © 2000 Cancer Research Campaign

**Keywords**: chromosomal in situ hybridization; multicentric renal cell tumours; chromosomal aberrations; tumour size, stage and grade

Some reports on renal cell tumours (RCTs) have shown evidence of multicentricity and/or bilaterality, in addition to various genetic and morphologic changes (Mukamel et al, 1988; Cheng et al, 1991; Henn et al, 1993; Zbar et al, 1994; Kletscher et al, 1995; Koolen et al, 1998). Although the incidence of multiple sporadic RCTs in the same kidney was estimated by Schubert (1984) to be 2–4%, prospective analysis of 100 radical tumour nephrectomy specimens showed 16% incidence (Kletscher et al, 1995). Tumours of different histology may be combined within one kidney (Kovács and Hoene, 1987; Delahunt and Eble, 1997). Many studies have shown various genetical changes in multicentric RCTs (Kovács and Hoene, 1987; Kovács et al, 1989; Henn et al, 1993; Zbar et al, 1994; Koolen et al, 1998); however, these studies used short-term cell culture, which, because of possible selection artefacts may not give accurate reflection of the cytogenetic characteristics of the RCTs being analysed (Wu et al, 1996). Furthermore, although chromosomal in situ hybridization (CISH) has been applied in the analyses of RCTs (El-Naggar et al, 1995; Wu et al, 1996; Siebert et al, 1998), few targeted studies have been performed on tumour nephrectomy specimens displaying simultaneously separate foci with different cytomorphologies.

*Received 24 March 1999*
*Revised 4 October 1999*
*Accepted 16 November 1999*

*Correspondence to:* BK Amo-Takyi

Conventional renal cell carcinomas (RCCs) reveal in more than 90% of cases loss of heterozygosity on 3p or monosomy 3 as the primary aberration (Kovács and Frisch, 1989; Meloni et al, 1992; Van den Berg et al, 1993; Amo-Takyi et al, 1998; Koolen et al, 1998). On the other hand, classical cytogenetics of multicentric papillary RCTs, whether adenomas or carcinomas, displayed common primary chromosomal aberrations in different foci, namely trisomy 17 and tri- or tetrasomy 7 (Kovács et al, 1991; Henn et al, 1993). These studies therefore associate specific genetic alterations with defined tumour cytomorphologies, as is reflected in the classification of RCTs (Störkel et al, 1997).

A common procedure in the cited reports involved the use of classical cytogenetics, a method which depends on tissue disaggregation and cell culturing, and allows statements about neither tissue morphology nor about relationships between tumours and their surrounding tissues. Conversely, CISH allows both determination of genetical changes and histological diagnosis (Neumann, 1987), and in situ verification of chromosomal anomalies by the histopathologist, whilst obviating the necessity for cell cultures or DNA extraction. It also permits concurrent assignment of chromosome changes to specific cellular or subcellular regions and judgement on their topological distributions (Gutenbach and Schmid, 1990).

In the event that the chromosomal aberrations demonstrated in the studies above are related specifically to conventional RCCs or papillary RCTs (adenomas and carcinomas), then each tumour focus in a multicentric disease should show the chromosomal aberrations associated with the particular cytomorphology of its

**Table 1** *P*-values after CISH for chromosomes 3, 7 or 17: comparison of signal distributions between the tumours and their surrounding normal kidney tissues

| Signals/cell | *P*-value | | | | | |
|---|---|---|---|---|---|---|
| | Chromosome 3 | | Chromosome 7 | | Chromosome 17 | |
| | NK vs CC | NK vs PT | NK vs CC | NK vs PT | NK vs CC | NK vs PT |
| 1 | 0.0042* | 0.2696 | 0.5832 | 0.0211 | 0.7227 | 0.0182 |
| 2 | 0.0001 | 0.1834 | 0.0009 | 0.0012 | 0.0011 | 0.0020 |
| 3 | – | 0.0006[a] | 0.0011[a] | 0.0020[a] | 0.0002 | 0.0001[a] |
| 4 | – | 0.0023[a] | 0.0042[a] | 0.0011[a] | 0.0022 | 0.0041[a] |

[a] Relevant *P*-values. Significant value: $P \leq 0.05$; –: not performed; NK = normal kidney tissue; CC = conventional RCCs; PT = papillary RCTs.

cells. In this study, we explored whether losses or gains of chromosomes 3, 7 or 17 could be demonstrated in each tumour focus of archived, formalin-fixed, paraffin-embedded sections of sporadic multicentric RCTs (blocks aged 1–6 years), using CISH. This was to demonstrate and validate the use of the CISH method in determining specific chromosomal aberrations in RCTs. We further sought for differences in chromosomal aberrations in relation to tumour size, stage, grade, intratumoral heterogeneity and also concordance or discordance of chromosomal changes between different foci in a specimen.

### MATERIALS AND METHODS

Two hundred and thirty-nine nephrectomy cases with sporadic RCTs were analysed for multicentricity. Thirty-four (14%) of them contained multiple tumours. Fifteen cases with 34 foci (16 conventional carcinomas, and 18 papillary RCTs (11 carcinomas and seven adenomas)) were analysed with CISH, using centromere-specific α-satellite probes for chromosomes 3, 7 or 17. To study the relationships between tumour size, malignancy, grade, differentiation and chromosomal aberrations, the 15 cases were selected to include adenomas and carcinomas, large- and small-sized tumours, high- and low-grade tumours, and cases (nine out of 15) with both conventional carcinomas and papillary tumours (adenomas and/or carcinomas) which were spatially separated by normal renal tissues.

### Histological procedure

Sections of the formalin-fixed, paraffin-embedded tumour specimens were stained in haematoxylin and eosin, and histological classification was performed as recommended by Störkel et al (1997) and Srigley et al (1997). Papillary tumours were considered adenomas when they were less than 5 mm, and did not fit into category I (Srigley et al, 1997); and all conventional tumours, irrespective of size, were classified as carcinomas.

### CISH

CISH was performed on paraffin sections of each tumour focus and surrounding normal renal tissues as described previously (Amo-Takyi et al, 1998), using the three chromosomes mentioned. Briefly, the 5- to 6-μm thin paraffin-embedded sections were mounted on sialinized slides and incubated at 60°C for 2 h. The sections were dewaxed in two changes of xylene, followed by two treatments in 100% ethanol immediately after the incubation. Proteinase K (Sigma, St Louis, MO, USA; 20 μg $ml^{-1}$ in phosphate-buffered saline (PBS)) digestion was performed in a humidified chamber at 37°C for 2 min. The sections were rinsed immediately in PBS and incubated with RNAase (Boehringer Mannheim, Germany; 40 μg $ml^{-1}$ in standard saline citrate) in a humidified chamber at 37°C for 1 h, post-fixed in 4% (w/v) paraformaldehyde in PBS for 3 min, rinsed in PBS and dehydrated in a graded ethanol series (50%, 70%, 90%, 100%) each for 3 min at room temperature and air-dried for 10 min. Ten microlitres of the DNA probes (digoxigenin-labelled α-satellite DNA probes for chromosomes 3, 7 or 17, concentration 1 μg 100 $\mu l^{-1}$, Oncor, Gaithersburg, MD, USA) per slide were pipetted into Eppendorf tubes and denatured in a water bath at 95°C for 5 min and immediately transferred onto ice for 2 min. The sections were also denatured separately in distilled autoclaved water at 95°C for 5 min and immediately transferred onto ice for 2 min. The sections were then allowed to air dry. Previously prepared hybridization mix (30 μl of 2 × hybridization buffer; Gibco-BRL, Life Technologies, Eggenstein, Germany and 40 μl of 10% (w/v) dextran sulphate) was added to the denatured DNA probes and applied onto each section, covered with coverslips, denatured for 10 min at 90°C, placed on ice for 2 min and incubated overnight in a humidified chamber at 37°C. Stringency washings with 60% (v/v) formamide in 2 × SET[27] (50 mM Tris–HCl, 300 mM sodium chloride (NaCl), 2 mM EDTA and 0.5% Triton X-100) at 37°C were performed at the end of the incubation. The sections were subsequently blocked with a solution containing bovine serum albumin, followed by application of alkaline phosphatase-labelled digoxigenin antibody (Boehringer Mannheim, Germany), and incubated at 37°C in a humidified chamber for 45 min. The hybrids formed were detected with nitroblue tetrazolium and 4-bromo-5-chloro-3-indolylphosphate (Gibco-BRL, Life Technologies, Eggenstein, Germany) for 18 h, counterstained in 1% methyl green in aqueous solution, and mounted permanently in eukitt. Hybridization signals were counted in 450 cell nuclei (running mean) for each chromosome. Internal controls were performed on normal renal tubular epithelial cells.

### Statistical analysis

The Wilcoxon rank sum test was used to examine the frequency distribution of hybridization signals. Comparisons of individual cases were performed with the Kruskal–Wallis test. Wilcoxon 2-Sample test was used to analyse the combined number of hybridization signals (1, 2, 3 etc.). Simple statistics and Pearson's correlation coefficients were also applied to establish the differences in chromosomal aberrations between small (≤ 1 cm) and large (> 1–15 cm) tumours, and between conventional carcinomas

 *© 2000 Cancer Research Campaign*

**Table 2** *P*-values after CISH for chromosomes 3, 7 or 17: comparison of signal distributions between conventional RCCs and papillary RCTs

| | ***P*-value** | | |
|---|---|---|---|
| **Signals/cell** | **Chromosome 3** | **Chromosome 7** | **Chromosome 17** |
| 1 | 0.0002[a] | 0.0114 | 0.0315 |
| 2 | 0.0010 | 0.3955 | 0.5508 |
| 3 | 0.1809 | 0.1158 | 0.0133[a] |
| 4 | 0.8482 | 0.0519 | 0.7974 |

[a]Relevant *P*-values. Significant value: $P \leq 0.05$.

**Table 3** Demographic details of the patients, including tumour sizes and differentiation.

| | **Solitary RCTs** | **Multicentric RCTs** |
|---|---|---|
| No. of cases | 205 | 34 (14%) |
| Male:female ratio | 1.4:1 | 2:1 |
| Male average age (years) | 59.4 | 60.1 |
| Female average age (years) | 62.5 | 67 |
| Tumour sizes (range) | 4 mm–15 cm | < 2 mm–15 cm |
| Differentiation | | |
| Conventional RCC | 83% | 47% |
| Papillary RCTs | 5% | 53% |
| Chromophobe RCTs | 2% | – |
| Oncocytoma | 10% | – |

**Table 4** Relationship of chromosomal findings with histological grade and stage in 16 conventional RCCs

| **Case number** | **Cell type** | **Tumour size (cm)** | **Histological grade** | **Tumour stage** | **Chromosomal findings** |
|---|---|---|---|---|---|
| 1 | a:clear | 15 | 3 | 3b | **Monosomy 3** |
| | b:clear | 0.5 | 2 | – | Disomy 3 |
| 2 | a:clear | 3 | 3 | 3a | Disomy 3 |
| 4 | a:clear | 8.5 | 2 | 2 | Disomy 3 |
| | b:clear | 0.8 | 3 | – | **Monosomy 3** |
| | c:clear | 0.8 | 1 | – | Disomy 3 |
| 5 | a:clear | 7 | 3 | 3a | Disomy 3 |
| | b:clear | 6 | 3 | – | Disomy 3 |
| 6 | a:clear | 4 | 2 | 2 | Disomy 3 |
| 7 | a:clear | 2 | 2 | 2 | Disomy 3 |
| 8 | a:clear | 3 | 2 | 2 | Disomy 3 |
| 9 | a:clear | 8 | 2 | 3b | Disomy 3 |
| | b:clear | 0.4 | 1 | – | Disomy 3 |
| 12 | a:clear | 13 | 2 | 2 | **Monosomy 3** |
| 13 | a:clear | 2.7 | 2 | 2 | Disomy 3 |
| 14 | a:clear | 4.5 | 1 | 2 | Disomy 3 |

a, b and c denote different tumour foci in the same kidney specimen; –, not done. Tumour staging was only performed for the biggest tumour in each specimen. Relevant findings are set in **bold** face.

and papillary RCTs as well. Significance was set at $P \leq 0.05$. Relevant *P*-values are marked with asterisks (*). Other irrelevant but significant values are caused by a shift in the equilibrium as soon as defined hybridization signals, i.e. 1, 2, 3 signals etc. outweigh. The *P*-values are summarized in Tables 1 and 2.

### Interpretation of results

Chromosomal changes in the normal renal tubular epithelial cells served as reference in defining the aberrations. Statistical evaluation of earlier work in our laboratory (unpublished data) revealed that 40% ± 5% of the nuclei in normal renal tubular epithelial tissues of 5- to 6-μm thickness display one hybridization signal after CISH. This stems from artefacts caused by truncation of some cell nuclei during tissue sectioning. Accordingly, a display of two hybridization signals of a diploid chromosome in at least 55% of the cell nuclei in a normal tissue of 5- to 6-μm thickness, even in the face of maximal truncations (45%), is viewed as disomic (normal). Hence, an increase of the upper value (45%) by at least 2D (D = +5%) indicated a tendency to a monosomy of the chromosome under investigation. Therefore tissues with 45% + 3D% or more one-hybridization signals of a particular chromosome were diagnosed as having acquired monosomies of that chromosome. Conversely, irrespective of the chromosome examined, a maximum of 5% (D) nuclei in 5- to 6-μm thick sections of normal tissues show three signals per nucleus after CISH. A trisomy was hence diagnosed only when D increased by at least twice (i.e. trisomy: ≥ 15% three hybridization signals). Cell subpopulations were considered when more than 5%, but less than 15% of the tumour cell nuclei revealed three or more copies of a chromosome.

### RESULTS

### Histomorphology

Table 3 gives detailed demographic information, including tumour differentiation and sizes. Thirty-four cases (14%) revealed multicentric RCTs. Each multicentric case contained at least two tumours. Most of the solitary cases were carcinomas, of which ten demonstrated extensive metastases at the time of clinical presentation. The smallest solitary RCT (a 4 mm adenoma) was detected incidentally in the course of an operation for urothelial carcinoma.

### Chromosomal aberrations determined by CISH

Normal renal tubular epithelial and surrounding stromal cells displayed mostly two hybridization signals, or sometimes one

*© 2000 Cancer Research Campaign*

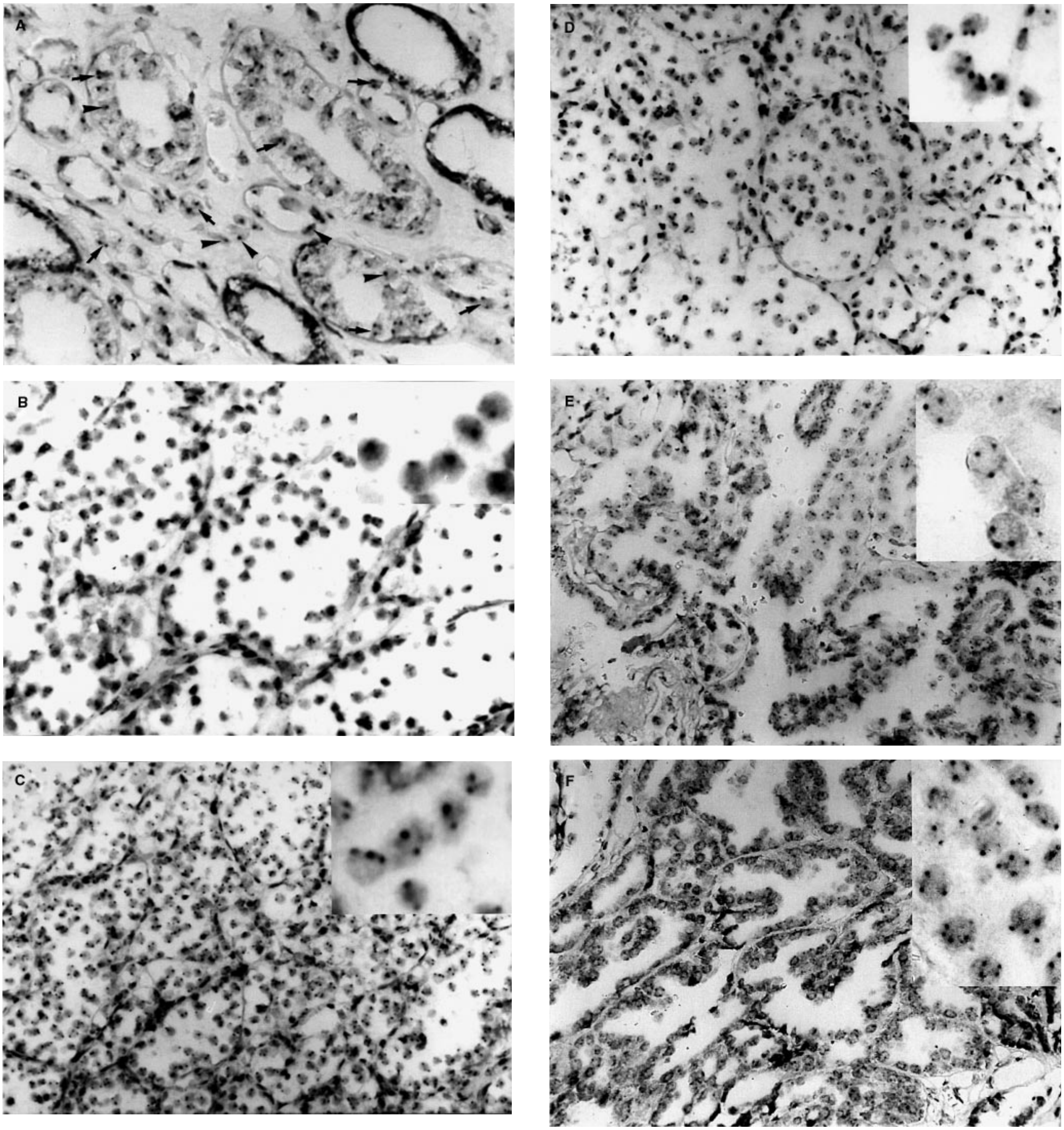

**Figure 1** (**A**) Normal renal tubular epithelial and surrounding stromal cells after CISH for chromosome 3. Like all subsequent hybridizations, it was counterstained in 1% methyl green. The cells display either one (arrowheads) or two hybridization signals (arrows). Chromosomes 7 and 17 also showed similar results. (**B**) A representative section of conventional RCC after CISH for chromosome 3, revealing mostly only one copy of the chromosome per nucleus (insert). (**C**) Conventional RCC after CISH for chromosome 7. As shown in the insert, most cells show two, and a few three hybridization signals. (**D**) Conventional RCC after CISH for chromosome 17, displaying mostly two copies (insert). (**E**) Overview of papillary RCC after CISH for chromosome 17. Insert shows magnified tumour nuclei with the characteristic three copies per nucleus. (**F**) Overview of papillary RCC after CISH for chromosome 7. Insert displays enlarged tumour nuclei with the characteristic three or four copies of chromosome 7 per nucleus.

hybridization signal (truncated nuclei), irrespective of the chromosome examined (Figure 1A).

Three conventional RCCs sized 8 mm, 13 cm and 15 cm respectively, exhibited monosomy 3 (Figure 1B, Table 4), and 13 conventional RCCs were disomic. Three conventional RCCs had trisomy 7 and cell subpopulations with four copies of chromosome 7. The remaining 13 conventional RCCs had disomy 7 (Figure 1C); however, they also showed various cell subpopulations with

*© 2000 Cancer Research Campaign*

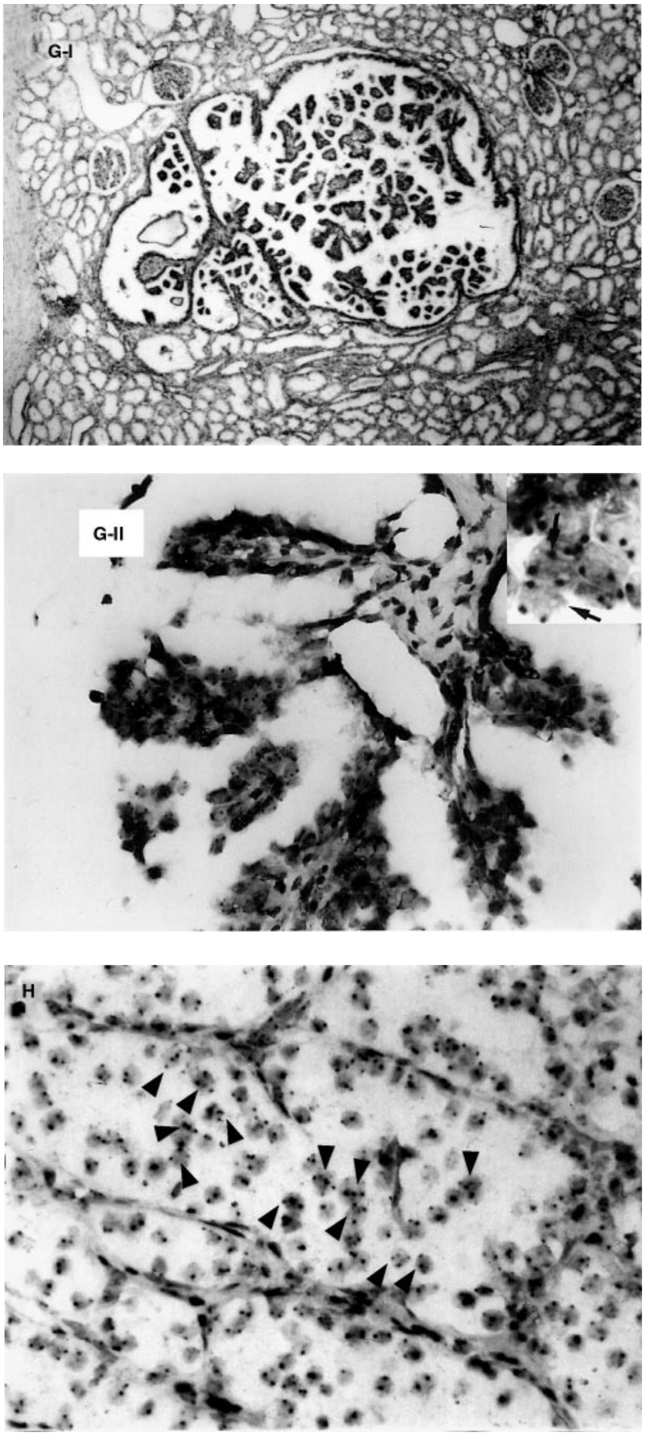

**Figure 1** (continued) (**G-I**) A small papillary renal adenoma; (**G-II**) A section of the adenoma after CISH for chromosome 17, revealing the characteristic three copies of the chromosome in some nuclei (arrows; insert). (**H**) Conventional RCC after CISH for chromosome 17 showing intratumoral heterogeneity. Note the focal cluster of cells with three copies (arrowheads); in contrast, the surrounding cells predominantly have two copies of the chromosome

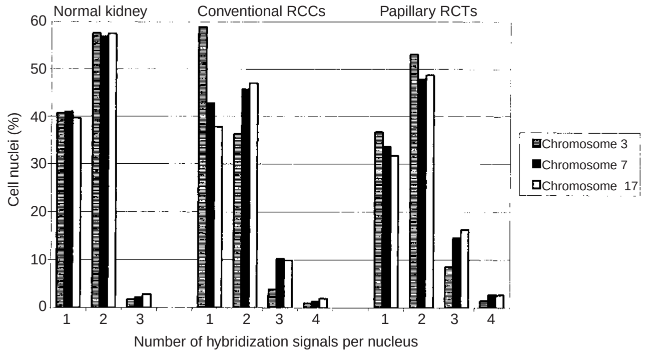

**Figure 2** Mean frequency distribution of signals after CISH. Roughly 40% of the normal kidney cells show one hybridization signal irrespective of the probe used. An average of 59% conventional RCC nuclei display a copy of chromosome 3, and an approximated 15% papillary RCT nuclei reveal three copies of chromosome 17. The number of nuclei with three copies of chromosome 7 are higher in both conventional RCCs and papillary RCTs than in the normal renal tissues

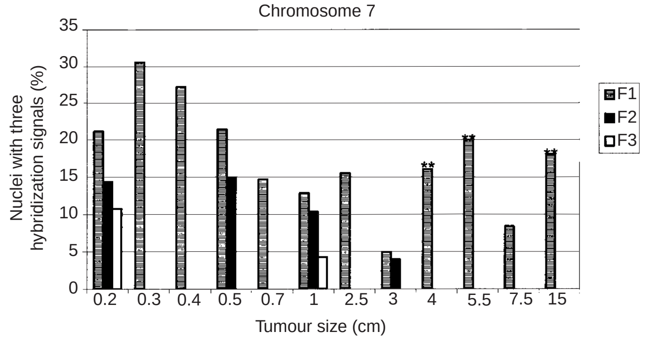

**Figure 3** Graphical presentation of multicentric papillary RCTs of different sizes, after CISH for chromosome 7. F1, F2 and F3 denote separate tumour foci in a specimen. Apart from three cases (**), there is some reduction of the copy numbers with tumour growth three (5.62–12.27%) and four (0–5.09%) copies of chromosome 7, respectively, as shown in the insert of Figure 1C. All 16 conventional RCCs were disomic for chromosome 17 (Figure 1D), nevertheless, there were cell subpopulations with three (6.94–13.77%) and four (0–4.57%) hybridization signals of chromosome 17 respectively.

Fourteen papillary RCTs (both adenomas and carcinomas) revealed trisomy 17 (Figure 1E). Three further small papillary RCTs (≤ 1 cm) displayed 14.49%, 14.68% and 14.86% cells, respectively, with three copies of chromosome 17. Ten papillary RCTs had tri- or tetrasomy 7 (Figure 1F). Even small papillary adenomas such as in Figure 1G-I (4 mm) revealed the characteristic trisomy 17 (Figure 1G-II). The results are summarized in Figures 2–5.

### DISCUSSION

The 14% sporadic multicentric RCTs observed in this study, and the 16% reported by Kletscher et al (1995) indicate a higher frequency of sporadic multicentric RCTs than the 2–4% assumed by Schubert (1984). Neither the tumour differentiation nor the chromosomal aberrations detected with CISH were related to patient age or gender. The differentiation of the solitary tumours were similar to those reported by others (Kovács, 1990; Linehan et al, 1995); however, the multicentric disease showed 47%

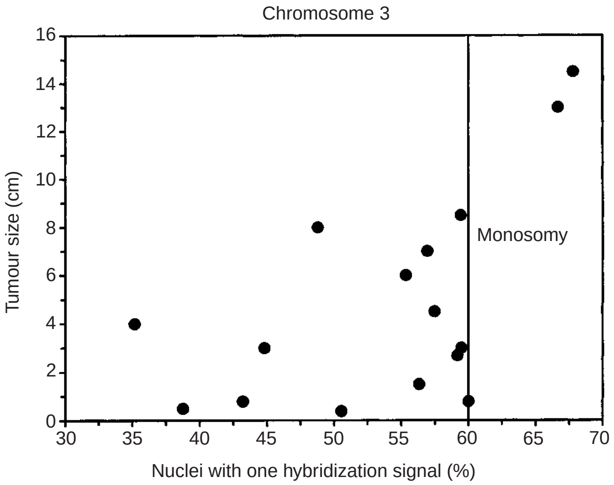

**Figure 4** A diagrammatical presentation of chromosomal aberrations in the 16 multicentric conventional RCCs. Monosomy 3 begins at 60%

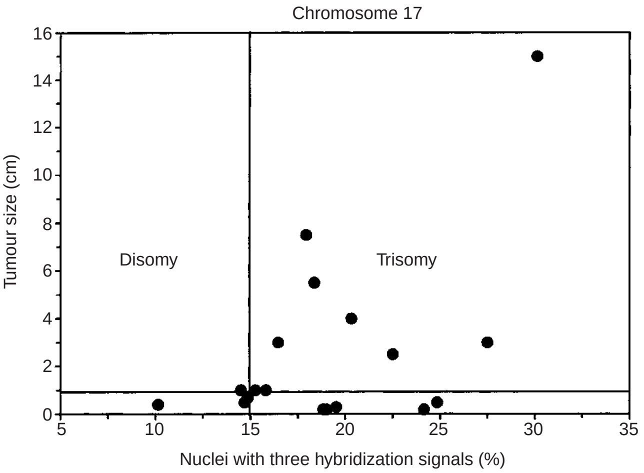

**Figure 5** A pictorial presentation of chromosomal aberrations in the 18 multicentric papillary RCTs. Tumours < 1 cm are considered small. Trisomy 17 starts from 15%

conventional RCCs (against 83% conventional RCCs in the cases with solitary RCTs) and 53% papillary RCTs (against 5% in the solitary RCTs). This demonstrates that papillary RCTs are liable to multicentricity, a fact which is of clinical importance.

### Chromosomal aberrations: multicentric conventional RCCs

Only three of the 16 conventional RCCs, sized 8 mm (G3), 13 cm (pT2, G2) and 15 cm (pT3b, G3), respectively, and all either high-grade or in advanced disease stages, exhibited monosomy 3. Hence, although monosomy 3 is characteristic of conventional RCCs (Kovács and Frisch, 1989; Amo-Takyi et al, 1998) heedless of size, its cytogenetical diagnostic value is limited, and can only be found in advanced- and/or high-grade tumours. Three conventional RCCs had trisomy 7 and disomy 17 and the rest were disomic for both chromosomes 7 and 17; however, the latter also showed various cell subpopulations with three or four copies of either chromosomes. Furthermore, comparison of the different foci in the same specimen was uninformative in the conventional RCCs.

Intratumoral heterogeneity was also clearly evident in all the tumours. There were clusters of compact, tubular or singular cell arrangements with higher copy numbers of all three tested chromosomes compared with the surrounding tumour cells (Figure 1H). This observation which is duly documented as subpopulations in this report, vividly demonstrates the heterogeneity of cell populations in a piece of tumour tissue. This brings to light that the end diagnosis whether mono-, di-, or trisomy etc. irrespective of the chromosome examined, is in fact, a subsumation of the actual chromosomal events taking place in a piece of tumour tissue. Consequently, intratumoral heterogeneity can not be demonstrated in studies which use classical cytogenetics or DNA cytometric analysis of tissues to detect aberrations or ploidy statuses due to the obligatory tissue disaggregation associated with both methods. Evidently, tissue disaggregation not only incapacitates histomorphological assessment, but also can lead to inaccurate genetic results since some heterogenous cell subpopulations may be missed at the time of tissue sampling.

No significant differences were observed in chromosomal aberrations between small and big conventional RCCs. Trisomy 7, a consistent abnormality in RCTs (Hogemann et al, 1994), and likewise increased copies of chromosome 17 appear to be expressions of general chromosomal instability in conventional RCCs in the course of tumour progress, and do not reflect specific cytogenetic changes.

It is histologically not possible to differentiate between primary tumours and their metastases based on tumour sizes alone. Again, a small tumour is not de facto metastasis of a bigger one, since a new tumour focus can develop even in the presence of a bigger one. The resolution of this question in cases 1, 4, 5 and 9 may have been provided by clonality analysis. Unfortunately, all four cases occurred in men, and hence, the well-known clonality analysis based on the pattern of X-chromosome inactivation (Tamura et al, 1998) was inapplicable. Clonality analysis was, nevertheless, not considered in those cases showing separate foci with different cytomorphologies, since no relationships could be envisaged. However, analyses are underway to clarify the clonality of some multifocal female RCTs, and the findings will be the subject of our next paper.

### Chromosomal aberrations: multicentric papillary RCTs

Fourteen of 18 papillary RCTs, both adenomas and carcinomas, revealed trisomy 17, without limitation to size or grade. Three further papillary RCTs had high numbers of cells with three hybridization signals: 14.49%, 14.68% and 14.86%, respectively, and were assumed to be incipient for trisomy 17 (see Figure 5). The results generally indicate a tumour type with a cytogenetic aberration unparalleled to other RCT subtypes. Apparently, all the three tumours incipient for trisomy 17 and the disomic tumour (10.14% cells with three copies of chromosome 17), are small tumours (≤ 1 cm). This presents a unique cytogenetic character of papillary RCTs, which though not restricted to size, showed an inclination to increased copy numbers with growing tumour size; showing a near significant $P$-value of 0.055. Moreover, the papillary RCTs were more susceptible to developing tri- or tetrasomy 7 compared to conventional RCCs.

 © 2000 Cancer Research Campaign

Despite the fact that tri- or tetrasomy 7 were common to papillary RCTs in general, the percentage of cells with three or four copies in the smaller papillary tumours, on average 19.36%, were comparably higher than those of the bigger papillary RCTs, which had a mean value of 11.84%. This implies that whereas smaller papillary RCTs, mostly adenomas, had overt tendencies to acquiring extra copies of chromosome 7, some copies got lost with continued tumour growth. This reduction, which was also observed in one small (2 mm) histologically grade 3 papillary tumour, probably reflects malignant transformation. Interestingly, focal areas of the papillary RCTs that showed clear cytoplasms, also revealed trisomies 17 and 7, meaning that growth pattern in papillary RCTs as it seems, might play a more dominant role than can possibly be ascribed to the morphological changes in the cytoplasm.

Comparison of different papillary RCT foci in the same specimen showed a general tendency of the bigger tumours to acquire three copies of chromosomes 3 and 17. This was generally reflected in near-significant $P$-values of 0.054 for trisomy 3 and 0.055 for trisomy 17 respectively. Moreover, as was observed in conventional RCCs, there were also heterogenous cell subpopulations in both papillary adenomas and papillary RCCs. Further, there was generally concordance of the basic chromosomal aberrations in the different foci in each specimen, i.e. the papillary tumours whether adenomas or carcinomas, revealed trisomy 17, in addition to di- or trisomy 7.

One small (2 mm) papillary tumour was not only histologically grade 3, but also showed disomy 7, which appeared in this report to be associated with malignancy. Consequently, this report does not support a strict differentiation of papillary adenomas from papillary RCCs based on whether or not 5 mm has been exceeded. We believe that both the histological grade and genetical changes should be considered, or else strict adherence to size alone may lead, in our opinion, to a false sense of security.

### Tissue thickness and truncation artefacts in the nuclei

The use of cell cultures or methods which disaggregate tissues by more or less maintaining the integrity of the individual cells, e.g. whole cell suspensions, cause minimal damage to cell membranes and hence, minimal loss of genetic material from the cell nuclei. However, such methods incapacitate histomorphological assessment, and therefore analysis of phenomena like intratumoral heterogeneity.

Nucleic acids in fixed tissue sections, i.e. the target DNAs, have to be unmasked and made available to the labelled DNA probe to be examined. The mainstay of this process is proteolytic digestion, which removes components of the cell nucleus and cytoplasm to allow probe access (Herrington, 1998). Thicker sections may not only be difficult to assess histomorphologically, but also may either need more proteolytic enzyme to make the target accessible to the probe and therefore increase the danger of overdigestion, or may be underdigested; in which cases there will be either loss of genetic material or suboptimal probe penetration. Thinner sections, on the other hand, may lead to overdigestion, and hence, destruction of cell and tissue architecture. This, thus, leads to loss of target DNA and morphological detail. In both these situations, there will be relative failure of the hybridization reaction as probe and target DNA are not brought together under optimal conditions (Herrington, 1998).

We initially performed the CISH, using sections of 3- to 4-μm thickness, just as in routine histology, for optimal morphologic assessment. In tissues of 3- to 4-μm thickness, ≥ 60% of the cell nuclei revealed one hybridization signal. Considering, however, that the average RCT nucleus has a diameter of about 7 μm, we found that the overrepresentation of one hybridization signal resulted from truncation artefacts, since with 3- to 4-μm sections we were only assessing about 40–60% of the nuclear DNA in each cell. Consequently tissue sections of 5- to 6-μm thickness were used. This allowed by optimal preservation of morphology and target accessibility to probe, evaluation of about 70–90% of the nuclear DNA per cell. In a word, selection of thin sections without regarding the average nuclear size of the cells in question may produce a biased high number of truncated cell nuclei, and inaccurate results.

The multicentric RCTs provided a good basis for assessing the chromosomal alterations in each focus whatever their cytomorphologies, in excellently preserved tissue morphology. Additionally, information pertaining to the tumour and its surrounding tissue was also obtainable. The method allowed a distinction of normal kidney tissues from conventional RCCs or papillary RCTs, and conventional RCCs from papillary RCTs by revealing different cytogenetic characters. It enabled assessment of heterogeneity of the tumours with respect to ploidy of individual cells in a single tumour, and underlined its potential in retrospective genetical characterization of paraffinized tumour tissues.

In conclusion, monosomy 3, although not restricted to tumour size, appears to be limited to high-grade and/or advanced conventional RCCs. Whereas the number of cells with three copies of chromosome 17 in papillary RCTs seem to increase with tumour growth, bigger tumours of the same subtype showed lesser numbers of cells with three copies of chromosome 7, possibly reflecting malignant transformation. These results, as it appears, might suggest that specific chromosomal alterations, in particular monosomy 3 in conventional RCCs and loss of chromosome 7 in papillary RCTs, since both were associated with histologically high-grade and/or advanced tumours, might be of value in predicting disease outcome.

### REFERENCES

Amo-Takyi BK, Handt S, Gunawan B, Hollweg H-G and Füzesi L (1998) A cytogenetic approach to the differential diagnosis of metastatic clear cell renal carcinoma. *Histopathology* **32**: 436–443

Cheng WS, Farrow GM and Zincke H (1991) The incidence of multicentricity in renal cell carcinoma. *J Urol* **146**: 1221–1223

Delahunt B and Eble JN (1997) Papillary renal cell carcinoma: a clinicopathologic and immunohistochemical study of 105 tumors. *Mod Pathol* **10**: 537–544

El-Naggar AK, Hurr K, Tu ZN, Tucker SL, Swanson D and Hsu PH (1995) Interphase cytogenetics of renal cortical neoplasms. Correlation with DNA ploidy by flow cytometry. *Am J Clin Pathol* **104**: 141–149

Gutenbach M and Schmid M (1990) Determination of Y chromosome aneuplodie in human sperm nuclei by nonradioactive in situ hybridization. *Am J Hum Genet* **46**: 553–558

Henn W, Zwergel T, Wullich B, Thönnes M, Zang K and Seitz G (1993) Bilateral multicentric papillary renal tumors with heteroclonal origin based on tissue-specific karyotype instability. *Cancer* **72**: 1315–1318

Herrington CS (1998) Demystified … in situ hybridisation. *J Clin Pathol: Mol Pathol* **51**: 8–13

Hogemann I, Bock S, Heppner P and Petrides PE (1994) Cytogenetic and growth factor gene analysis of a renal cell carcinoma cell line. *Cancer Genet Cytogenet* **78**: 175–180

© 2000 Cancer Research Campaign 

Kletscher BA, Qian J, Bostwick DG, Andrews PE and Zincke H (1995) Prospective analysis of multifocality in renal cell carcinoma: influence of histological pattern, grade, number, size, volume and deoxyribonucleic acid ploidy. *J Urol* **153**: 904–906

Koolen MI, van der Meyden AP, Bodmer D, Eleveld M, van der Looij E, Brunner H, Smits A, van den Berg E, Smeets D and Geurts van Kessel A (1998) A familial case of renal cell carcinoma and a t(2;3) chromosome translocation. *Kidney Int* **53**: 273–275

Kovács G (1990) Guest editorial. Application of molecular cytogenetic techniques to the evaluation of renal parenchymal tumors. *J Cancer Res Clin Oncol* **116**: 318–323

Kovács G and Hoene E (1987) Multifocal renal cell carcinoma: a cytogenetic study. *Virchows Arch A* **412**: 79–82

Kovács G and Frisch S (1989) Clonal chromosome abnormalities in tumor cells from patients with sporadic renal cell carcinomas. *Cancer Res* **49** 651–659

Kovács G, Brusa P and De Riese W (1989) Tissue-specific expression of a constitutional 3;6 translocation: development of multiple bilateral renal-cell carcinomas. *Int J Cancer* **43**: 422–427

Kovács G, Füzesi L, Emanuel A and Kung H (1991) Cytogenetics of papillary renal cell tumors. *Genes Chromosomes Cancer* **3**: 249–255

Linehan MW, Lerman MI and Zbar B (1995) Identification of the von Hippel-Lindau (VHL) gene. Its role in renal cancer. *JAMA* **273**: 564–570

Meloni Aurelia M, Bridge Julia and Sandberg Avery A (1992) Reviews on chromosome studies in urological tumors. I. Renal Tumors. *J Urol* **148**: 253–265

Mukamel E, Konichezky M, Engelstein D and Servidio C (1988) Incidental small renal tumors accompanying clinically overt renal cell carcinoma. *J Urol* **140**: 22–24

Neumann R (1987). Die Technik der Nukleinsäure-hybridisierung und ihre Bedeutung für diagnostische Fragestellungen. *Naturwissenschaften* **74**: 125–133

Schubert GE (1984) Niere und ableitende Harnwege. In: *Pathologie*, Vol **3**, Remmele W (ed), pp 95. Springer: Berlin

Siebert R, Jacobi C, Matthiesen P, Zuhlke-Jenisch R, Potratz C, Zhang Y, Stockle M, Kloppel G, Grote W and Schlegelberger B (1998) Detection of deletions in the short arm of chromosome 3 in uncultured renal cell carcinomas by interphase cytogenetics. *J Urol* **160**: 534–539

Srigley JR, Hutter RVP, Gelb AB, Henson DE, Kenney G, King BF, Raziuddin S and Pisansky TM (1997) Current prognostic factors – renal cell carcinoma. Workgroup No. 4. Communication: Union Internationale Contre le Cancer (UICC) and the American Joint Committee on Cancer (AJCC). *Cancer* **80**: 994–996

Störkel S, Eble JN, Adlakha K, Amin M, Blute ML, Bostwick DG, Darson M, Delahunt B and Iczkowski K (1997) Classification of renal cell carcinoma. Workgroup No. 1. Communication: Union Internationale Contre le Cancer (UICC) and the American Joint Committee on Cancer (AJCC). *Cancer* **80**: 987–989

Tamura M, Fukuya T, Murakami T, Uehara S and Yjima A (1998) Analysis of clonality in human endometriotic cysts based on evaluation of X chromosome inactivation in archival formalin-fixed, paraffin-embedded tissue. *Lab Invest* **78**: 213–218

Van den Berg E, Van der Hout AH, Oosterhuis JW, Störkel S, Dijkhuizen T, Dam A, Zweers HMM, Mensink HJA, Buys CHCM and De Jong B (1993) Cytogenetic analysis of epithelial renal cell tumors: relationship with a new histopathological classification. *Int J Cancer* **55**: 223–227.

Wu SQ, Hafez GR, Xing W, Newton M and Messing E (1996) The correlation between the loss of chromosome 14q with histologic tumor grade, pathologic stage, and outcome of patients with nonpapillary renal cell carcinoma. *Cancer* **77**: 1154–1160

Zbar B, Tory K, Merino M, Schmidt L, Glenn G, Choyke P, Walther MM, Lerman M and Linehan WM (1994) Hereditary papillary renal cell carcinoma. *J Urol* **151**: 561–566

 *© 2000 Cancer Research Campaign*
